# The "Referee" Children's Dinner Fund

**Published:** 1907-11-23

**Authors:** 


### THE *REFEREE* CHILDREN'S DINNER FUND.

SIR,—Will you kindly permit us to appeal through your columns to the charitable public on behalf of the underfed children attending our elementary schools?

For many years past the *Referee* Children's Dinner Fund, the London Schools Dinner Association, and other Associations have rendered valuable assistance in collecting and distributing funds. With these Associations the Council is in close connection, and every effort is being made to bring the Council, the Associations, and the schools into such relationship as will result in a highly efficient organization for relieving distress.

The winter will soon be upon us, when distress must inevitably increase, and, in order to meet the needs of the children, the Council is anxious that at least 15,000*l.* should be raised.

The Council considers that it would be a great misfortune if voluntary contributions, which have hitherto met the demand, should fail to continue to do so. If, however, the response is not adequate this winter, there will probably be no alternative in the winter of 1908-9 but to resort to the rates.

The Council has voted a sum for equipment and appliances, and will place every convenience at the disposal of the Associations, through the medium of the Children's Care Committees which have been appointed for every necessitous school. These Committees will make all possible effort to insure the most careful discrimination in the selection of those children who are really in need of help.

Contributions may be sent to :—

(1) The *Referee* Children's Dinner Fund, Hon. Treasurer, Mrs. Burgwin, 147 Brixton Road, S.W.

(2) The London Schools Dinner Association, Hon. Treasurer, the Right Hon. Lord Kinnaird, 1 Pall Mall East, S.W.

(3) Any of the other Associations co-operating with the Council.

Or to one of us the undersigned :—

HENRY PERCY HARRIS, Chairman of the London County Council, 98 Gloucester Terrace, Hyde Park, W.

JOHN T. TAYLOR, Chairman of the Education Committee, 19 Woodchurch Road, Hampstead, N.W.

E. A. H. JAY, Chairman of the Sub-Committee on Underfed Children, Tower House, Woolwich.

